# Attentional Bias to Beauty with Evolutionary Benefits: Evidence from Aesthetic Appraisal of Landscape Architecture

**DOI:** 10.3389/fpsyg.2018.00071

**Published:** 2018-02-07

**Authors:** Wei Zhang, Xiaoxiang Tang, Xianyou He, Shuxian Lai

**Affiliations:** ^1^School of Architecture, State Key Laboratory of Subtropical Building Science, South China University of Technology, Guangzhou, China; ^2^Guangdong Engineering & Technology Research Center for Modern Architecture Design, South China University of Technology, Guangzhou, China; ^3^Guangdong Key Laboratory of Mental Health and Cognitive Science, Center for Studies of Psychological Application, School of Psychology, South China Normal University, Guangzhou, China; ^4^Preschool Education Guidance Center of Tianhe District, Public Kindergarten of Guangzhou Government, Guangzhou, China

**Keywords:** aesthetic appraisal of landscape architecture, evolutionary aesthetics, attentional bias, faster orienting of attention, delayed attentional disengagement

## Abstract

Substantial evidence suggests that beauty is associated with the survival and reproduction of organisms. Landscape architecture is composed of a series of natural elements that have significant evolutionary implications. The present study used one pilot material ratings and three experiments to examine the mechanisms of aesthetic appraisals of landscape architecture. The results confirmed that landscape architecture elicited a sense of beauty and captured visual attention more easily than other types of architecture during explicit aesthetic rating task (Experiment 1) and implicit aesthetic perception task (dot-probe paradigm, Experiment 2). Furthermore, the spatial cueing paradigm revealed that response latencies were significantly faster for landscape architecture than non-landscape architecture on valid trials, but there was no significant difference in this contrast on invalid trials at 150-ms stimulus onset asynchrony (SOA, Experiment 3a). At 500-ms SOA (Experiment 3b), participants responded significantly faster for landscape architecture on valid trials, but reacted significantly slower for landscape architecture on invalid trials. The findings indicated that the beauty of landscape architecture can be perceived implicitly, and only faster orienting of attention, but not delayed disengagement of attention was generated at early stages of the processing of landscape architecture. However, the attentional bias at later stages of attentional processes may be resulted from both faster orienting of attention and delayed disengagement of attention from landscape architecture photographs.

## Introduction

Imagine you are beholding landscape architecture (e.g., classical gardens of Suzhou, China) either in person or through photographs. How you feel about it? Studies widely show that human beings experience higher levels of positive emotion, relaxation and life satisfaction when they are exposed to natural scenarios or viewing photographs or paintings of landscapes with green spaces and gardens (Bratman et al., 2012; Guitart et al., 2012; Zhang et al., 2014). Meanwhile, humans also display a strong preference for and give high aesthetic appraisals of landscapes and landscape architectures (Lindemann-Matthies and Brieger, 2016).

According to the viewpoint of evolutionary aesthetics, perception of beauty is an evolving behavior in environmental adaption, and it is strongly associated with survival and reproduction of organisms (Killin, 2013; Seghers, 2015). A number of studies have demonstrated that participants preferred representational paintings which include elements of water and green plants (Dutton, 2003). Moreover, real scenarios with visual elements such as water sources, green plants, and open space (Dutton, 2009), and audio clips with sound of water were more easily judged as beautiful (see Seghers, 2015). This aesthetic preference could be interpreted as an adaptive value that might be important for the survival and reproduction of organisms. Following the logic of evolutionary aesthetics and evolutionary psychology, we asked the question of whether landscape architecture would elicit a sense of beauty more easily than other types of architecture because it is a type of architecture that consists of elements (e.g., water, green plants, and rockeries) that have significant evolutionary implications.

Previous evolutionary aesthetics researches also suggested that beauty (mainly referred to facial beauty) or stimuli with strong reproductive values (e.g., the whole body, the waist and the hips) can effectively capture and hold attention under constrained viewing conditions (Confer et al., 2010; Lu and Chang, 2012). In the pioneering work, Maner et al. (2007a,b) adopted the dot-probe task, a widely used paradigm for investigating attentional bias, and found participants’ reaction to target categorizations was significantly slower when the targets followed high attractive face images. Similar findings were observed in the spatial endogenous cuing task, which showed the attractive faces significantly lengthened target location judgment (Sui and Liu, 2009). Further eye tracking and neurophysiological evidence revealed that attractive faces, especially attractive opposite-sex faces perceived by male participants, could capture more visual attention and fixation (Valuch et al., 2015), and elicit larger amplitudes of the P2 component, which have been found to be related to implicit selective attention (van Hooff et al., 2011). Based on these findings, we raised the second question of the present study: does similar attentional bias also operate in the processing of landscape architecture due to its dual properties of beauty and evolutionary implications?

In addition, if landscape architecture stimuli can bias attention allocation, the psychological mechanisms underlying the attentional bias effect on landscape architecture remain unclear. Previous studies have demonstrated that the mechanisms of attentional bias and orienting cannot be considered as fully automatic (for a review see Santangelo and Spence, 2008), and further researches also adopted the spatial cueing paradigm and identified that there are two main components of attentional bias, faster orienting and delayed disengagement of attention (Posner, 1980; Posner and Petersen, 1990; Vogt et al., 2008; Langley et al., 2011; Hu et al., 2014; Vromen et al., 2014; Harrison and Woodhouse, 2016), which correspond to early and later attentional processing, respectively. In this paradigm, participants need to judge whether the dot (target) is presented on the left or right side of the screen. The presence of target is preceded by a cue, either at the same location (valid trial) or the opposite location (invalid trial). In addition to valid and invalid cues, some researchers create a set of neutral trials that serve as baseline condition (Peterson and Gibson, 2011) or filler trials (Vogt et al., 2008). If attentional bias is driven by faster attentional orienting, ration times (RTs) in detecting the dot on valid trials will decrease. In contrast, delayed disengagement of attention is evidenced by longer response latencies on invalid trials.

In assessing the aesthetic preference for landscape architecture during explicit and implicit aesthetic perception and judgment, we used photographs of Chinese classical landscape architecture and classical non-landscape architecture to reduce the confounding effects of design philosophy and prestige. Experiment 1 used the explicit aesthetic rating task to investigate whether landscape architecture can elicit a sense of beauty more easily than other types of architecture. Experiment 2 adopted the dot-probe paradigm (implicit aesthetic perception task) to examine whether the beauty of landscape architecture can be perceived implicitly. That is, whether participants display an obvious attentional bias toward landscape architecture. Finally, we conducted Experiments 3a and 3b with the spatial cueing paradigm to identify the cognitive components underlying this attentional bias at different stages of cognitive processing.

## Pilot Material Ratings

### Method

#### Participants

Thirty college students (17 females), aged between 18 to 24 years (mean age = 19.60, *SD* = 1.61) were recruited locally to take part in the material ratings. None of the participants had special training in art or architecture. The pilot material ratings and the following experiments were carried out in accordance with the recommendations of Institute Ethics Committee, South China Normal University with written informed consent from all participants. All participants gave written informed consent in accordance with the Declaration of Helsinki. The protocol was approved by the Institute Ethics Committee, South China Normal University.

#### Materials and Task

The 102 colored architectural photographs (51 classical landscape architectures) were selected from public Internet sources. Participants were instructed to rate these photographs on a 5-point scale, in terms of (*i*) the aesthetic quality of the architectural photographs, (*ii*) the complexity of the architectural photographs, and (*iii*) the familiarity of the architecture in the photographs.

### Results

Based on the ratings, 24 colored photographs of classical landscape architectures and 24 colored photographs of classical non-landscape architectures were selected as experimental materials.

Results of the pilot material ratings confirmed that there was no significant difference between classical landscape architecture photographs (3.65 ± 0.26) and classical non-landscape architecture photographs (3.65 ± 0.22) in the aesthetic quality of architectural photographs, *t*(46) = 0.02, *p* = 0.986. There was no significant difference between the two sets of materials in the complexity of architectural photographs (3.22 ± 0.21; 3.19 ± 0.23), *t*(46) = 0.56, *p* = 0.580. Meanwhile, no significant difference was found for the familiarity of the architecture between the classical landscape architecture photographs (1.10 ± 0.06) and the classical non-landscape architecture photographs (1.14 ± 0.19), *t*(46) = -1.00, *p* = 0.324.

Furthermore, in order to rule out the effect of visual saliency in attentional capturing, we used the Graph-Based Visual Saliency (GBVS) model implemented by Harel et al. (2006) to compute saliency score for each experimental stimulus. Results of saliency score revealed no significant difference between the two sets of experimental materials, *t*(23) = 1.11, *p* = 0.281.

## Experiment 1

### Method

#### Participants

Forty healthy college students (25 females), right-handed, with normal or corrected-to-normal vision, aged between 18 and 25 years (mean age = 20.68, *SD* = 1.66) were recruited locally and paid for their participation. Participation was restricted to individuals who had no special experience in art or architecture.

#### Materials

Twenty-four colored photographs of classical landscape architecture and 24 colored photographs of classical non-landscape architecture were used based on the selections of the pilot material ratings. Stimuli were edited and standardized with Adobe Photoshop CS6 in terms of removing elements of persons, adjusting to be approximate luminance and color saturation, adjusting to be equal size and resolution within a rectangular ‘window’ sized 300 × 200 pixel, and centring the photographs on a 600 × 400 pixels gray background (25% gray scale).

#### Design

This experiment involved a single within-subjects factor (types of architecture: classical landscape architecture vs. classical non-landscape architecture) with 24 trials of each condition. The dependent variables were aesthetic rating scores and RTs of the aesthetic judgment task to architecture photographs.

#### Procedure

The testing procedure consisted of 96 trials (with one repetition of the 48 experimental materials), which were randomly divided into four experimental blocks. During the testing procedure, participants were asked to rate the aesthetic score of architecture photographs with a five-point rating scale (1 for not beautiful at all, 5 for very beautiful), by pressing one of the five buttons on the keyboard. Each trial of the testing procedure consisted of the following events: a red fixation point was presented for 600 ms, followed by the experimental stimulus (with a maximum allowed reaction time of 3000 ms) and then a gray screen (800 ms) displaying as an inter-stimulus interval (ISI). There was a short resting period between each block. Prior to the testing procedure, participants were asked to perform 20 practice trials in a training session.

### Results and Discussion

Data for aesthetic rating scores and RTs deviating more than 2.5 standard deviations from the condition mean were removed from the data, resulting in the removal of 1.35% of the data of rating scores, and 1.88% of the data of RTs.

Mean values and standard errors of aesthetic rating scores and RTs in all conditions are shown in **Table 1**. One-way repeated-measures ANOVA with subjects as random effect were conducted on the aesthetic rating scores and revealed no significant differences between classical landscape architecture and non-landscape architecture photographs, *F*(1,39) = 1.55, *p* = 0.220, η^2^ = 0.04.

**Table 1:** Mean values and standard errors of aesthetic rating scores and RTs in all conditions.

	Landscape architecture	Non-landscape architecture
Aesthetic rating scores	3.85 ± 0.74	3.68 ± 0.63
Mean RTs	954 ± 178	1012 ± 249

Although there was no significant main effect of types of architecture on the aesthetic rating scores, which replicated the similar findings of the pilot study of material ratings, we still found significant differences in the RTs of aesthetic ratings, which revealed that response latencies were faster for classical landscape architecture photographs than for non-landscape architecture photographs, *F*(1,39) = 4.81, *p* = 0.034, η^2^ = 0.11.

In line with our hypothesis, Experiment 1 demonstrated a clear facilitated effect on perceiving the beauty of classical landscape architecture photographs during the explicit aesthetic rating task, which indicates that classical landscape architecture can elicit a sense of beauty more easily than other types of architecture. As an explicit aesthetic judgment task was used in Experiment 1, some strategies of responses would be developed. In Experiment 2, we used the dot-probe paradigm to investigate whether there was an attentional bias toward classical landscape architecture photographs, and we tried to provide preliminary evidence to verify that the beauty of classical landscape architecture can be perceived implicitly.

## Experiment 2

### Method

#### Participants

A different group of 32 healthy college students (20 females), right-handed, with normal or corrected-to-normal vision, aged between 18 and 24 years (mean age = 20.91, *SD* = 1.53) was recruited locally and paid for their participation. Participation was restricted to individuals who had no special experience in art or architecture.

#### Materials

The standardized colored photographs of classical landscape and non-landscape architecture were identical to those in Experiment 1. We also created two stimuli categories: The lower definition versions for the original colored photographs and the random digit stimuli. The lower definition versions were created in Adobe Photoshop CS6 and served as a comparison condition to rule out the effects of color preference. The random digit stimuli were used to reduce strategic monitoring on one side of the screen.

#### Design

Experiment 2 involved a 2 (dot-photograph locations: dot presented on the same side of classical landscape architecture vs. dot presented on the same side of classical non-landscape architecture) × 2 (types of stimuli: original version vs. lower definition version) factorial design. All variables were manipulated within subjects. The dependent variable was RTs of the judgment of dot location.

#### Procedure

**FIGURE 1:**
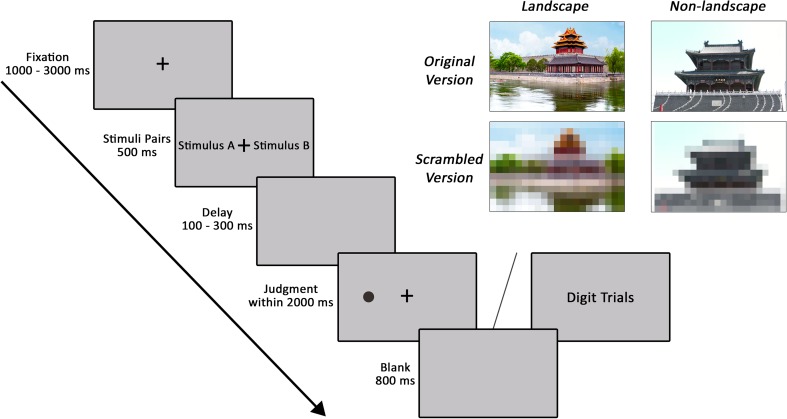
The experimental procedure of Experiment 2 is displayed on the left. Examples of classical landscape architecture and non-landscape architecture pairs are displayed on the right. In this figure, we used photographs we took instead of examples of stimuli in the experiment due to the Open Access Creative Commons licensing.

**Figure 1** illustrates the examples of stimuli and the experimental procedures for Experiment 2. Participants performed a dot-probe task that consisted of 108 presentation trials split into four 27-trial blocks (12 standardized colored landscape architecture and non-landscape architecture photograph pairs, 12 lower definition versions for the corresponding original colored photograph pairs, and 3 random digit trials). At the beginning of each trial, participants were presented with a fixation point with a variable duration, ranging from 1000 to 3000 ms. Then, in each experimental trial, one of the landscape architecture and non-landscape architecture photograph pairs or the corresponding lower definition version was presented for 500 ms. Following offset of the stimuli pairs, the screen was blank for 100–300 ms. After then, a black dot was presented at the center of the location where previously occupied by one of the two photographs for the maximum allowed reaction time of 2000 ms. In 50% of trials, the dot appeared on the side of landscape architecture photograph, and in the other 50% of trials, the dot appeared on the side of non-landscape architecture photograph. Participants were instructed to press one of the two buttons to indicate whether the dot was located on the left or right side of the screen. In each of 6 experimental trials, a random digit (catch trial) from 1 to 9 was presented at the center of the screen in order to reduce strategic monitoring on one side of the screen. Participants needed to respond with the space key within 2000 ms in the catch trial. The next trial started after an 800 ms inter-trial interval.

### Results and Discussion

**FIGURE 2:**
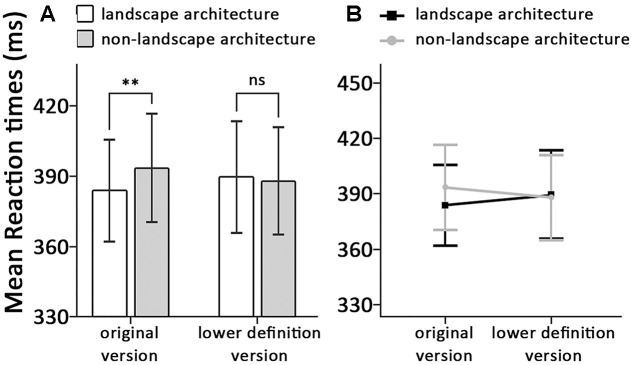
**(A)** Means and standard errors, **(B)** the interactions around correct reaction time, displayed as a function of the dot-probe locations and the types of stimuli, ^∗^*p* < 0.05, ^∗∗^*p* < 0.01.

Trials with error reaction, data for RTs deviating more than 2.5 standard deviations from the condition mean, and trials with RTs below 200 ms or above 2000 ms were removed as outliers, resulting in the removal of 2.28% of the data. Mean RTs in all conditions are shown in **Figure 2A**.

#### Error Rates

A two-way repeated-measures ANOVA with subjects as random effect conducted on error rates revealed no significant main effect of types of stimuli, *F*(1,31) = 0.49, *p* = 0.488, η^2^ = 0.02, and no significant main effect of dot-photograph locations, *F*(1,31) = 0.24, *p* = 0.625, η^2^ = 0.01. Moreover, no significant interaction between dot-photograph locations and types of stimuli was found, *F*(1,31) = 0.33, *p* = 0.572, η^2^ = 0.01.

#### Reaction Time

A two-way repeated-measures ANOVA with subjects as random effect conducted on RTs revealed no significant main effect of types of stimuli, *F*(1,31) < 0.01, *p* = 0.970, η^2^ < 0.01. A significant main effect of dot-photograph locations was found, *F*(1,31) = 5.00, *p* = 0.033, η^2^ = 0.14. Consistent with our predictions, we also found a significant interaction between dot-photograph locations and types of stimuli, *F*(1,31) = 7.34, *p* = 0.011, η^2^ = 0.19. Within the original version condition, we found response latencies for detecting the dot were significantly lower when it appeared on the same side of classical landscape architectures (384 ± 60) than when it appeared on the same side of classical non-landscape architecture (393 ± 64), *t*(31) = -3.00, *p* = 0.005, Cohen’s *d* = 0.50. However, the response latencies within the lower definition version condition revealed no significant difference between dot appeared on the same side of classical landscape architecture (390 ± 67) and dot appeared on the same side of classical non-landscape architecture (388 ± 64), *t*(31) = 0.81, *p* = 0.423, Cohen’s *d* = 0.15 (see **Figure 2B**).

Taken together, these results revealed a strong attentional bias effect for classical landscape architecture photographs, which suggest that participants attend to classical landscape architecture rather than non-landscape architecture photographs. To further investigate the psychological mechanisms underlying this attentional bias effect on classical landscape architecture photographs, Experiments 3a and 3b employed the spatial cueing paradigm by manipulating different levels of stimulus onset asynchronies (SOAs) to discuss whether the attentional bias of classical landscape architecture results from faster orienting of attention, delayed disengagement, or both.

## Experiment 3a

### Method

#### Participants

A total of 30 healthy college students (18 females), right-handed, with normal or corrected-to-normal vision, aged between 18 and 25 years (mean age = 20.30, *SD* = 1.71) were recruited locally. None of the participants had special experience in art or architecture and had ever participated in other experimental tasks. All participants were paid for their participation.

#### Materials

The standardized colored photographs of classical landscape and non-landscape architectures, the random digit stimuli were identical to those in Experiment 2. Because the preference of colors was ruled out in Experiment 2, we did not use the lower definition version as comparison conditions in Experiment 3a.

#### Design

Experiment 3a involved a 2 (types of architecture: classical landscape architecture vs. classical non-landscape architecture) × 2 (cue validity: valid vs. invalid) factorial design. All variables were manipulated within subjects. The dependent variable was RTs of the judgment of dot location.

#### Procedure

**FIGURE 3:**
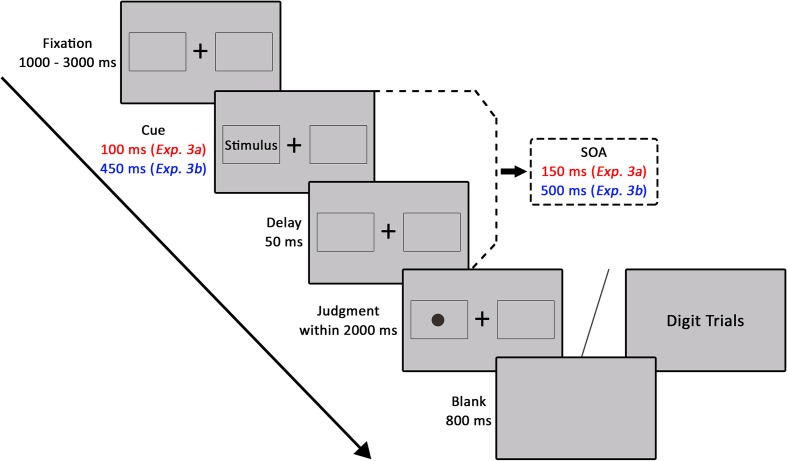
The experimental procedure of Experiments 3a and 3b.

The experimental procedure of a given trial was displayed in **Figure 3**. All stimuli were presented on a gray background. On each trial, a black fixation cross (at the center of screen) and two peripheral squares (in the left and right of the horizontal center of screen) were presented randomly from 1000 to 3000 ms. Preceding the onset of another 50-ms fixation period, cues consisting of architecture photographs were presented within one of the peripheral squares for 100 ms (SOA of 150 ms). The target (a black dot) was presented at the center of one of the peripheral squares for the maximum allowed reaction time of 2000 ms. There were two validity conditions: in 50% of trials, the target was presented on the same side of the classical landscape or non-landscape architecture photograph (valid), and in the other 50% of trials, the target was presented on the opposite side of the photograph (invalid). Participants were instructed to press one of the two buttons to indicate whether the dot was located on the left or right side of the screen. Similar to Experiment 2, in each of 6 experimental trials, a random digit from 1 to 9 was presented at the center of the screen in order to reduce strategic monitoring on one side of the screen. Participants needed to respond with the space key within 2000 ms in the catch trial. The next trial started after an 800 ms inter-trial interval.

### Results and Discussion

**FIGURE 4:**
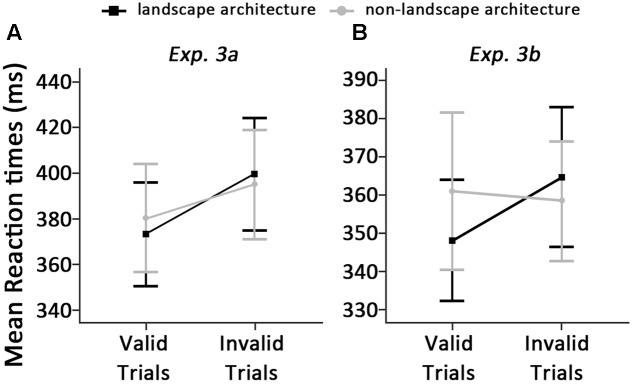
Means and standard errors around correct reaction time, displayed as a function of types of architecture and cue validity in Experiments 3a **(A)** and 3b **(B)**.

The standards of outlier elimination were the same as Experiment 2, which resulted in the removal of 3.68% of the data. Mean RTs in all conditions are shown in **Figure 4A**.

#### Error Rates

A two-way repeated-measures ANOVA with subjects as random effect conducted on error rates revealed no significant main effect of types of stimuli, *F*(1,29) = 0.27, *p* = 0.610, η^2^ = 0.01. Significant main effect of cue validity was found, *F*(1,29) = 7.59, *p* = 0.010, η^2^ = 0.21, which revealed error rates were higher on invalid trials than valid trials. Moreover, no significant interaction between cue validity and types of stimuli was found, *F*(1,29) = 0.21, *p* = 0.647, η^2^ = 0.01.

#### Reaction Time

In order to identify whether the attentional bias of landscape architecture resulted from faster orienting of attention, delayed disengagement, or both, mean RTs for dot detection in valid and invalid trials were compared by conducting a two-way repeated-measures ANOVA with subjects as random effect. If attentional bias is driven by faster attentional orienting of classical landscape architecture photographs, response latencies for detecting the dot on valid trials of landscape architecture photographs will decrease, whereas difficulty disengaging from classical landscape architecture photographs will show in slower responses on invalid trials.

Results showed no significant main effect of types of architecture, *F*(1,29) = 0.35, *p* = 0.557, η^2^ = 0.01. A significant main effect of cue validity was found, *F*(1,29) = 22.52, *p* < 0.001, η^2^ = 0.44. We also found a significant interaction between types of architecture and cue validity, *F*(1,29) = 8.79, *p* = 0.006, η^2^ = 0.23, which revealed that RTs were significantly faster for detecting the dot when classical landscape architecture photographs served as cues (373 ± 61) compared to those when the cues were classical non-landscape architecture photographs (380 ± 64) on valid trials, *t*(29) = -2.39, *p* = 0.023, Cohen’s *d* = 0.45. However, there was no significant difference in this contrast on invalid trials (399 ± 66 vs. 395 ± 64, between classical landscape architecture and non-landscape architecture photographs, respectively), *t*(29) = 1.65, *p* = 0.110, Cohen’s *d* = 0.25. This indicates only faster orienting of attention to landscape architecture photographs but not delayed disengagement of attention at early stages of attentional processes. As 150-ms SOA was used in Experiment 3a, which could be regarded as the early stages of attentional processing. In Experiment 3b, the cognitive components of attentional bias at the later stages of visual processing will be discussed.

## Experiment 3b

### Method

#### Participants

A different group of 30 healthy college students (19 females), right-handed, with normal or corrected-to-normal vision, aged between 18 to 25 years (mean age = 20.67, *SD* = 2.14) was recruited locally. None of the participants had special experience in art or architecture and had ever participated in other experimental tasks. All participants were paid for their participation.

#### Materials

The experimental materials were identical to those in Experiment 3a.

#### Design and Procedure

The experimental design and procedure were similar to those in Experiment 3a. Differently from Experiment 3a, the cues consisted of architecture photographs in Experiment 3b were presented within one of the peripheral squares for 450 ms (SOA of 500 ms).

### Results and Discussion

The standards of outlier elimination were the same as Experiment 2, which resulted in the removal of 3.82% of the data. Mean RTs in all conditions are shown in **Figure 4B**.

#### Error Rates

Similar to Experiment 3a, a two-way repeated-measures ANOVA with subjects as random effect conducted on error rates revealed no significant main effect of types of stimuli, *F*(1,29) = 2.73, *p* = 0.109, η^2^ = 0.09. Significant main effect of cue validity was found, *F*(1,29) = 8.37, *p* = 0.007, η^2^ = 0.22, which revealed error rates were higher on invalid trials than valid trials. Moreover, no significant interaction between cue validity and types of stimuli was found, *F*(1,29) = 1.61, *p* = 0.214, η^2^ = 0.05.

#### Reaction Time

A two-way repeated-measures ANOVA with subjects as random effect conducted on RTs revealed neither a main effect of types of architecture, *F*(1,29) = 1.28, *p* = 0.267, η^2^ = 0.04, nor a main effect of cue validity, *F*(1,29) = 1.85, *p* = 0.185, η^2^ = 0.06. However, we found a significant interaction between types of architecture and cue validity, *F*(1,29) = 8.23, *p* = 0.008, η^2^ = 0.22, which revealed that participants responded significantly faster in detecting the dot when classical landscape architecture photographs served as cues (348 ± 43) compared to those when the cues were classical non-landscape architecture photographs on valid trials (360 ± 55), *t*(29) = -2.28, *p* = 0.030, Cohen’s *d* = 0.44. On invalid trials, participants detected the dot significantly slower when classical landscape architecture photographs served as cues (365 ± 49) compared to those when the cues were classical non-landscape architecture photographs (359 ± 42), *t*(29) = 2.28, *p* = 0.030, Cohen’s *d* = 0.41. These results indicated that the attention bias at later stages of attentional processes may be resulted from both faster orienting of attention and delayed disengagement of attention from classical landscape architecture photographs.

## General Discussion

The present results revealed strong aesthetic preference and attentional bias for landscape architecture than for other types of architecture. In Experiment 1, we found a clear facilitated effect on perceiving the beauty of landscape architecture during explicit aesthetic rating task. In Experiment 2, 3a and 3b, we used implicit aesthetic perception tasks (under constrained viewing conditions) and found that landscape architecture was prioritized in visual processing. These findings suggest that the beauty of landscape architecture may attract more visual attention in both explicit and implicit aesthetic perception. Moreover, this attentional bias emerged quickly at the early stages of attentional processing and was maintained endogenously during the later stages of visual processing.

These results are in line with previous findings that visual attention not only preferentially responds to the signals of threat-related and negative stimuli (Öhman and Mineka, 2001; Leutgeb et al., 2015), but also shows a bias toward stimuli with strong positive evolutionary implications (Maner et al., 2007a; Nummenmaa et al., 2011). As we created a lower definition version for each original colored photograph and found that the aesthetic preference and attentional bias for landscape architecture were present only in the original photograph condition but not in the lower definition version condition, the effect of color preference in capturing attention could be ruled out. More importantly, this argument is strengthened by the fact we balanced the visual saliency, which could not only increases the probability of recollection (Pedale and Santangelo, 2015; for a review see Santangelo, 2015), especially in the context-congruent information (Santangelo et al., 2015), but also attract gaze and attention, and further modulates the activation of visual-related regions, and dorsal and ventral attention systems during scene viewing according to previous studies (for a review see Itti and Koch, 2001; Nardo et al., 2011, 2014).

One possible explanation for our findings is that humans possess an innate preference for landscapes with water and green plants, which contain strong evolutionary implications (Dutton, 2003, 2009). As the main components of landscape architecture, water and green plants are necessary for human survival, the presence of landscape architecture may signal the potential benefits of living and reproduction, which may generate a greater sense of safety and facilitate aesthetic appraisal of landscape architecture according to the framework of evolutionary aesthetics (Killin, 2013; Seghers, 2015).

Again, the present study attempted to investigate two main components of attentional bias—faster orienting and delayed disengagement of attention—by adopting the spatial cueing paradigm. Evidence of faster orienting of attention was found for landscape architecture cues at both early and later stages of attentional processes. However, delayed disengagement of attention for landscape architecture cues was found only at later stages of attentional processes. The faster orienting of attention may serve as a reminder of the present stimulus which is relevant to one’s needs, and the delayed disengagement of attention is likely to act as an index for further information processing. Therefore, these findings were consistent with the Component Process Model (CPM), which proposed the early attentional process is responsible to detect whether the visual stimuli are relevant to the individual, and modulate subsequent attentional resources toward these stimuli (Sander et al., 2005). Taken together, it can be inferred that evolution may shape the attention for effective detection in visual environments, which makes individuals are more sensitive and responsive to landscape architecture cues.

In addition, Posner et al. (1985) first reported the ‘inhibition of return’ effect (IOR effect), revealing that responses were slower to the target at the cued location than at the uncued location when the SOA was longer than 300 ms. Researchers have argued that this IOR effect might serve as an adaptive mechanism to prevent reorienting to previously attended locations (Posner et al., 1985). However, our present study did not find the IOR effect in the 500-ms SOA condition. We assumed that the absence of the IOR effect may be attributed to the delayed disengagement of attention for landscape architecture cues. Previous studies have found that the more beautiful a stimulus, the more attention and the longer gaze it attracts (Lindell and Lindell, 2014; Leder et al., 2016). Therefore, we inferred that our beautiful landscape architecture photographs which composed of a series of natural elements with significant evolutionary implications could capture longer visual attention at the cued location, made it hard to drift back to the central fixation cross, and thus substantially reduce the IOR effect (Fox et al., 2002).

To sum up, the aesthetic preference and attentional bias for landscape architecture found in this study signal the adaptive significance of this type of architecture. More broadly, these findings lend preliminary evidence to the explanation that the sense of beauty for landscape architecture is rooted in evolution. Future research may focus on collecting additional evidence (e.g., eye tracking and event-related potentials) to further examine the time course during explicit and implicitly processing for the sense of beauty of landscape architecture. Another issue that may be addressed in subsequent research concerns the question of whether the effect of attentional bias is caused by some of the components or the overall configurational features of landscape architecture.

## Author Contributions

WZ designed the experiments and drafted the article; XT and XH revised this manuscript critically; SL for data pre-processing and analysis.

## Conflict of Interest Statement

The authors declare that the research was conducted in the absence of any commercial or financial relationships that could be construed as a potential conflict of interest.
